# Supplementary figures and images for: Costs and models used in the economic analysis of Total Knee Replacement (TKR): A systematic review
Source: PLoS One. 2023 Jul 25;18(7):e0280371. doi: 10.1371/journal.pone.0280371 (PMC10368258; doi:10.1371/journal.pone.0280371)

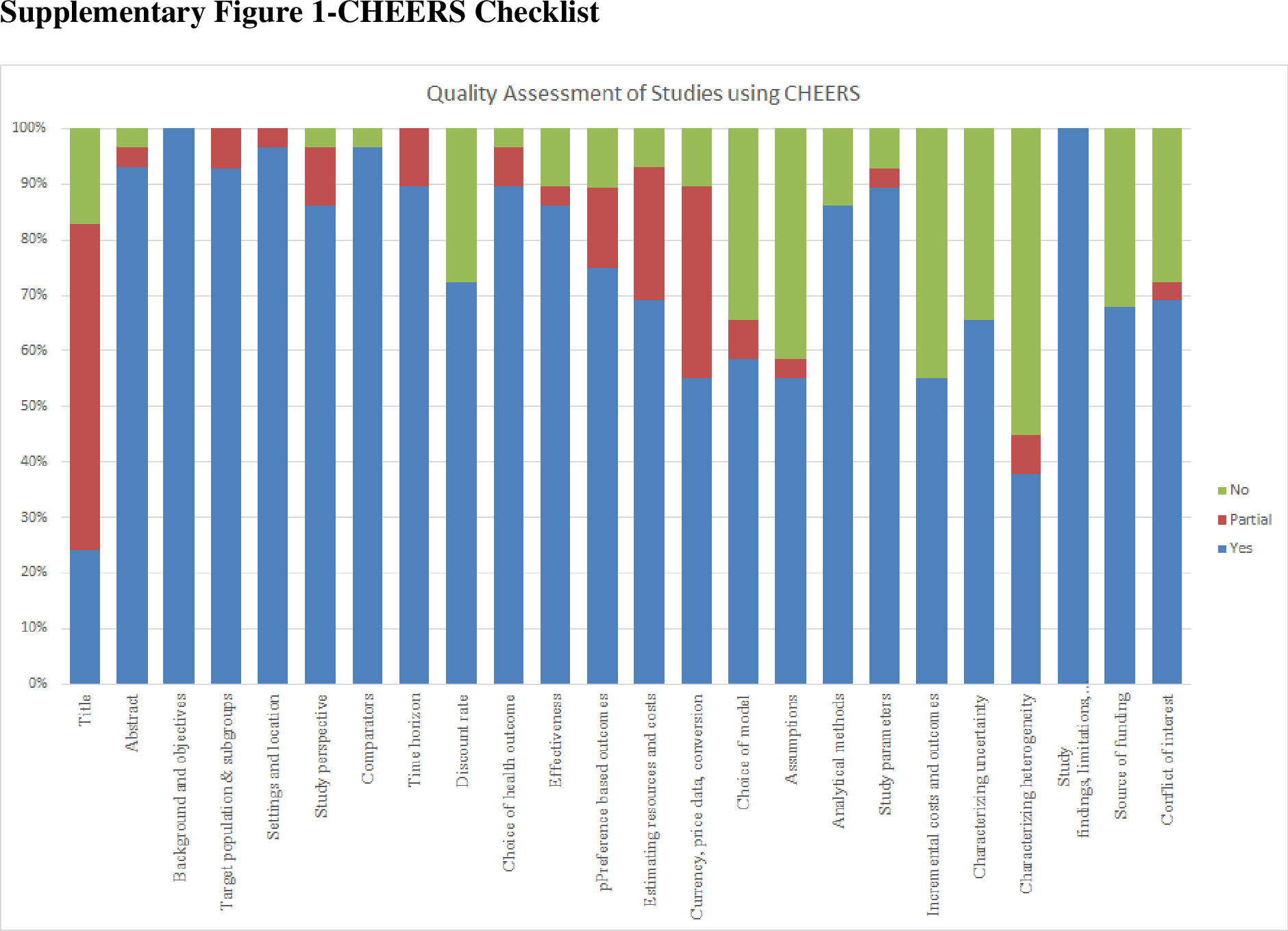

Supplement: S1 Fig — (TIF) [file pone.0280371.s002.tif]

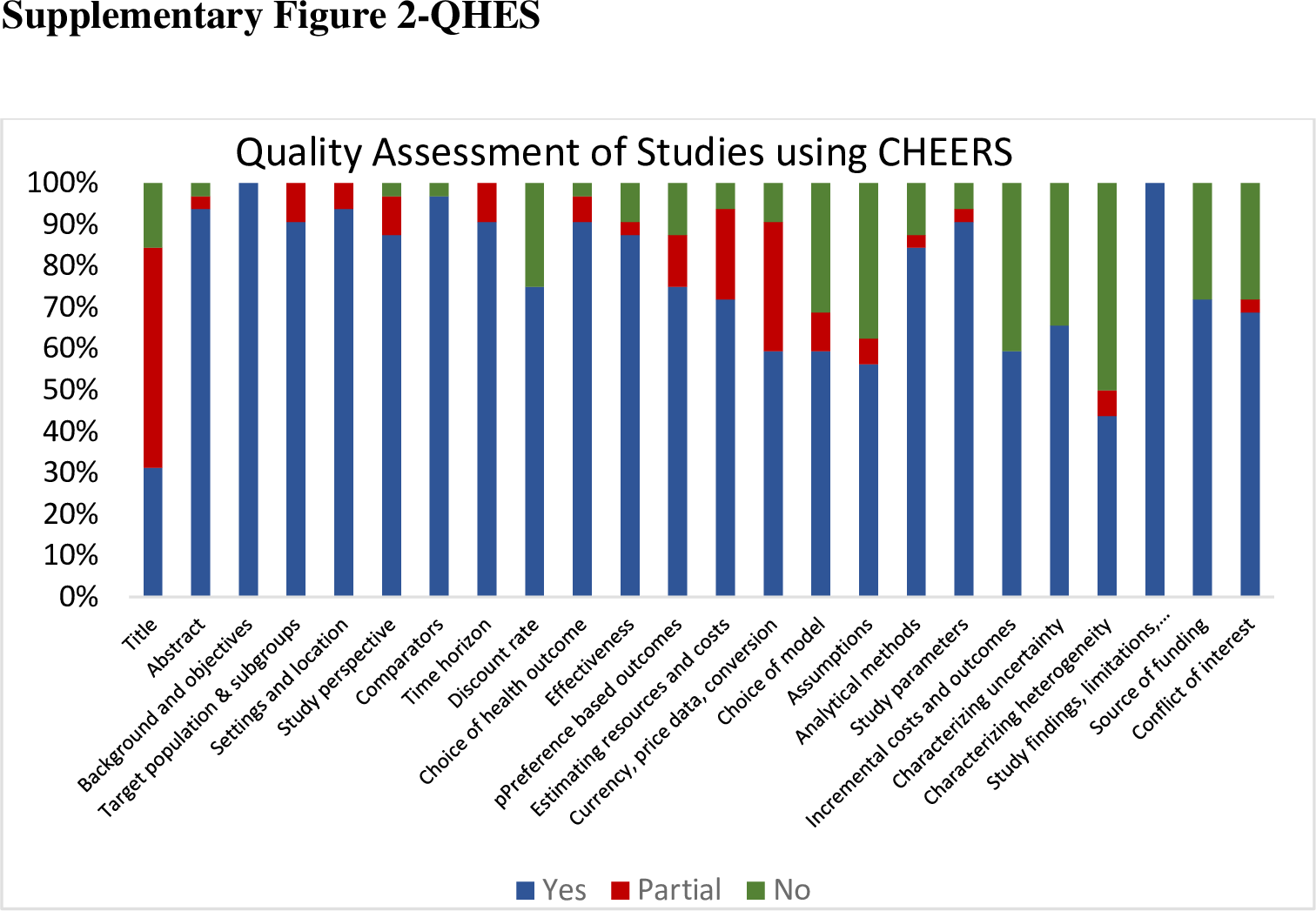

Supplement: S2 Fig — (TIF) [file pone.0280371.s003.tif]
