# Supplementary material for: Costs and models used in the economic analysis of Total Knee Replacement (TKR): A systematic review
Source: PLoS One. 2023 Jul 25;18(7):e0280371. doi: 10.1371/journal.pone.0280371 (PMC10368258; doi:10.1371/journal.pone.0280371)
Supplement: S1 Table — (DOCX) [file pone.0280371.s004.docx]

Supplementary Table 1: Models and Cost of TKR used across studies

| **Author Name** | **Year** | **Location** | **Transition probabilities** | **Models** | **Justification and scope of the Model** | **Perspective** | **Currency Metric** | **TKR Cost** | **Cost of TKR in 2021 (after adjusting CPI inflation)** | **COST of TKR in PPP $ in 2021** | **Health outcome measurement** | **Cost-effectiveness measurement** | **Willingness to pay threshold** |
| --- | --- | --- | --- | --- | --- | --- | --- | --- | --- | --- | --- | --- | --- |
| Kazarian et.al, | 2018 | USA | R | Markov Model | NR | Societal | USD | 17915 | 19332 | 19332 | QALY | ICER | $50,000 |
| Skou et.al | 2020 | Denmark | NA | Linear Regression Model | NR | Provider | EURO | 13149 | 13393 | 20066 | QALY | ICER | € 22665 |
| Ponnusamy et.al | 2018 | Canada | R | Markov Model | NR | Provider | USD | 18746 | 19902 | 19902 | QALY | ICER | $30,000 |
| Elmallah et al | 2017 | USA | NA | NA | NR | Payer | NA | NA | NA | NA | QALY, SF6D | ICER | $50,000 |
| Losina et.al | 2009 | USA | R | Markov Model | NR | Societal | USD | 20700 | 26145 | 26145 | QALY | ICER | NA |
| Cohen et.al | 2016 | USA | NA | NA | NR | Provider | USD | 8546 | 9648 | 9648 | NA | Cost comparison | NA |
| Waimann et.al | 2014 | USA | NA | NA | NR | Societal | USD | 24435 | 27968 | 27968 | MCID, WOMAC | ICER | $50,000 |
| Ferket et.al | 2017 | USA | R | Marginal Structural Model | R | Provider | USD | 16051 | 17744 | 17744 | QALY | ICER | $200000 |
| Higashi et.al | 2011 | Australia | NA | Discrete Simulation Model | R | Provider | AUD | 13640 | 16409 | 11403 | DALY | Cost per DALY averted, ICER | AUD 50,000/DALY |
| Jenkins et.al | 2013 | UK | NA | Decision Tree Model | NR | NC | GBP | 7313 | 8312 | 11994 | QALY | ICER | £20 000 to £30 000 |
| Krummenauer et.al | 2009 | Germany | NA | Logistic Regression Model | NR | Provider | EURO | 9549 | 11295 | 16923 | QALY | Median cost-effectiveness ratio per QALY gained | £30,000–£50,000 |
| Rasanen et. al | 2007 | Finland | NA | NA | NR | Provider | NA | NA | NA | NA | QALY | Cost-utility ratio | NA |
| Karuppiah et.al | 2008 | UK | NA | NA | NR | Provider | GBP | 16932 | 21914 | 31623 | KSS, WOMAC | Cost benefit | € 30,000 per QALY |
| Dakin et.al | 2012 | UK | NA | Regression Model, GLM | R | Provider | GBP | 6363 | 7398 | 10675 | QALY | ICER | £20000-£30 000/QALY |
| Rissanen et. al | 1997 | Finland | NA | NA | NR | Provider | NA | NA | NA | NA | 15D score | ICER | NA |
| Stan et.al | 2015 | Romania | NA | NA | NR | Provider | EURO | 9549 | 11159 | 16719 | QALY | Cost utility ratio | NA |
| Mari et.al | 2016 | France | R | Markov Model | NR | Patient | EURO | 6624 | 7038 | 10546 | QALY | Cost-utility ratio | €30,000 to €50,000 |
| Schilling et.al | 2017 | Australia | NA | Linear Regression Model | NR | Provider | AUD | 21553 | 23152 | 16089 | QALY | ICER | $50,000 |
| Bedair et. al | 2014 | USA | R | Markov Model | NR | Patient | NA | NA | NA | NA | Working days | Cost benefit | NA |
| George et.al | 2021 | India | R | Markov Model | NR | Patient | INR | 80000 | 80000 | 3457 | QALY | ICER | ₹25,000 to ₹50 000 |
| Ruiz et.al | 2013 | USA | R | Markov Model | NR | Societal | USD | 20635 | 24002 | 24002 | Working Days | Cost benefit | $50,000 |
| Karmarkar et.al | 2017 | USA | R | Markov Model | R | Patient | USD | 17662 | 19525 | 19525 | QALY | Cost-utility ratio | NA |
| Navarro et.al | 2008 | Spain |  | NA | NR | Provider | EURO | 6866 | 7946 | 11906 | QALY | ICER | $50,000 to $100,000 |
| Zicat et.al | 1993 | Canada | NA | NA | NR | Patient | NA | NA | NA | NA |  | Cost comparison | NA |
| Mahendira et.al | 2020 | Canada | NA | NA | NR | Patient | CAD | 9551 | 9875 | 7881 | WOMAC score | Cost benefit | NA |
| Gui et.al | 2019 | China | NA | NA | NR | Patient | Chinese Yean | 57941 | 59925 | 14311 | QALY, SF-36 | Cost benefit, cost utility | £20,000 to £30,000 |
| Lavernia et.al | 1997 | USA | NA | NA | NR | Provider | NA | NA | NA | NA | Quality of well-being Index | Cost per quality well year | NA |
| Lovald et.al | 2013 | USA | NA | Regression Model (GLM) | NR | Societal | USD | 11000 | 12795 | 12795 | NA | Incremental cost | NA |
| Peggy et.al | 2012 | Canada | NA | NA | NR | Provider | CAD | 10959 | 12754 | 10179 | QALY | ICER | NA |
| Wilson et.al | 2021 | New Zealand | R | Markov Model | R | Patient | NZD | 16,903 | 16903 | 11375 | QALY | ICER | $60,600 |
| Serikova-Esengeldina et.al | 2022 | Khazakhstan | R | NA | NA | Patient | USD | 5156.5 | 4848 | 4848 | QALY | ICER | $9812.39 |
| Chen et.al | 2021 | USA | R | OAPol | R | Provider | USD | 18955 | 18955 | 18955 | QALY | ICER | NR |
