# Supplementary material for: Costs and models used in the economic analysis of Total Knee Replacement (TKR): A systematic review
Source: PLoS One. 2023 Jul 25;18(7):e0280371. doi: 10.1371/journal.pone.0280371 (PMC10368258; doi:10.1371/journal.pone.0280371)
Supplement: S1 Appendix — (DOCX) [file pone.0280371.s005.docx]

Appendix: Search Strategies

The MESH terms for keywords of interest were identified and Boolean operators were used in conjunction to prepare the search parameter. The final search parameters used were:

(("arthroplasty, replacement, knee"[MeSH Terms] OR ("arthroplasty"[All Fields] AND "replacement"[All Fields] AND "knee"[All Fields]) OR "knee replacement arthroplasty"[All Fields] OR ("total"[All Fields] AND "knee"[All Fields] AND "arthroplasty"[All Fields]) OR "total knee arthroplasty"[All Fields]) AND ("cost benefit analysis"[MeSH Terms] OR ("cost benefit"[All Fields] AND "analysis"[All Fields]) OR "cost benefit analysis"[All Fields] OR ("cost"[All Fields] AND "effectiveness"[All Fields]) OR "cost effectiveness"[All Fields])) OR (("arthroplasty, replacement, knee"[MeSH Terms] OR ("arthroplasty"[All Fields] AND "replacement"[All Fields] AND "knee"[All Fields]) OR "knee replacement arthroplasty"[All Fields] OR ("total"[All Fields] AND "knee"[All Fields] AND "arthroplasty"[All Fields]) OR "total knee arthroplasty"[All Fields]) AND ("quality adjusted life years"[MeSH Terms] OR ("quality adjusted"[All Fields] AND "life"[All Fields] AND "years"[All Fields]) OR "quality adjusted life years"[All Fields] OR "qaly"[All Fields]))
