# Supplementary material for: Costs and models used in the economic analysis of Total Knee Replacement (TKR): A systematic review
Source: PLoS One. 2023 Jul 25;18(7):e0280371. doi: 10.1371/journal.pone.0280371 (PMC10368258; doi:10.1371/journal.pone.0280371)
Supplement: Attachment — Submitted filename: Response to Reviewer.docx [file pone.0280371.s006.docx]

## Rebuttal Letter to manuscript PONE-D-22-35337- [EMID: 8c30f2f8667e9c0a]:

Costs and models used in the economic analysis of Total Knee Replacement (TKR): A Systematic Review

Dear Editor,

I, on behalf of all the authors would like to thank you for your valuable comments and suggestions, which gave us the opportunity to improve the paper. In the revised manuscript, we have addressed all the issues raised. Moreover, in this document, I provide point by point response to queries raised by the journal. Comments are shown in bold font, followed by our answer/comment in normal font. The major corrections/changes in the manuscript are displayed in red font.

**1. Please ensure that your manuscript meets PLOS ONE's style requirements, including those for file naming. The PLOS ONE style templates can be found at**

[**https://journals.plos.org/plosone/s/file?id=wjVg/PLOSOne_formatting_sample_main_body.pdf**](https://journals.plos.org/plosone/s/file?id=wjVg/PLOSOne_formatting_sample_main_body.pdf) **and**

[**https://journals.plos.org/plosone/s/file?id=ba62/PLOSOne_formatting_sample_title_authors_affiliations.pdf**](https://journals.plos.org/plosone/s/file?id=ba62/PLOSOne_formatting_sample_title_authors_affiliations.pdf)**.**

We have revised the manuscript to meet PLOS ONE’s style requirements, including file naming according to the templates provided

**2. We note that the grant information you provided in the ‘Funding Information’ and ‘Financial Disclosure’ sections do not match.**

**When you resubmit, please ensure that you provide the correct grant numbers for the awards you received for your study in the ‘Funding Information’ section.**

We have clarified in detail, the fund receipt process for developing the manuscript. Department of health research (DHR), MoHFW, Government of India, has signed a MoU with IIPH Hyderabad to establish a regional resource center for conducting Health technology assessment (HTA).  This resource center as a part of its ongoing research conducted this literature review. The staff engaged in this activity were funded by the DHR.

**3. We note that the manuscript is reporting a meta-analysis on genetic association studies. We need you to provide us with additional information in relation to this meta-analysis; please complete the following checklist and upload it as a Supporting Information file with a file name “GAMA checklist”. The checklist can be downloaded here:** [**http://www.plos.org/wp-content/uploads/2013/05/meta-analysis-on-genetic-association-studies-form.docx**](http://www.plos.org/wp-content/uploads/2013/05/meta-analysis-on-genetic-association-studies-form.docx)**.**

The manuscript does not have a meta-analysis component, nor does it report on genetic studies. Hence, a GAMA checklist is not applicable to our study.

**Reviewer #1:**

**The study on ‘costs and models used in the economic analysis of Total Knee Replacement (TKR) – a systematic review’ is simple, clear and to the point. It is useful in devising best model for cost effectiveness that can be utilized in further studies. The review also highlights the point that low- and middle-income countries despite having limited number of studies also have lowest cost of TKR surgery. The cost extraction through PPP method is helpful in identifying the best medical tourism for TKR across different countries of the world.**

1. **References 7 and 9 are the same, repeated.**

Thank you very much for your detailed observation and we have made the necessary changes in the manuscript.

**Reviewer #2:**

**1. The study overall is well-structured and clearly written.**

**2. Google Scholar was not mentioned as a resource for literature in the methodology and abstract sections but appeared in the results section.**

We have changed as per the suggestions in all the sections.

**3. The discussions regarding the Markov model (Lines 334-342) were general and focused on the characteristics of the model. It would have been beneficial to have a more comprehensive discussion, specifically addressing why the Markov model is more applicable or appropriate for TKR.**

This is an important observation and in the revised manuscript, we have explicitly mentioned the merits of Markov model as this captures different health states when a person can move from one state to other and again reverses. When TKR surgery is done, the patient may transition from bad to good and further may return to a worse situation, Markov Model captures all these transitions and provides their ICER values.

We have elaborated on this in Lines 353-371.

**4. The definition of direct and indirect costs needs clarification. For example, in Line 257, the authors mentioned "cost of travel." If this refers to transportation costs covered by health insurance, it might fall under direct non-medical costs. It would be helpful to explain this in more detail, perhaps by providing a brief explanation in the text or adding footnotes to Table 1.**

We have clarified the definition of direct and indirect cost in Lines (261-264) as used by the manuscript under review. Importantly, direct cost includes cost of medicine, diagnostic services, cost of implant, doctor’s fee and other hospitalization related expenditure, and non-medical cost such as cost of food, travel related to hospital access. Indirect cost includes —income loss due to TKR and income loss of caretaker of the patient.

**5. The section pertaining to the cost data for TKR surgery is somewhat ambiguous (Line 288-296). Could you please clarify the reporting process for the cost of TKR surgery in this section? It's unclear if ancillary costs were factored into the reported cost or if it is strictly focused on the surgical expenses. Also, does the reported cost represent the price per surgery? Your clarification would greatly enhance our understanding of the data.**

We have added details on data collection of costs in Lines (299-302). Moreover, studies that collected costs from national database and secondary literature are cited as well.

**6. In Line 320-322, you mentioned that "The TKR surgery is also a cost-effective method of treatment for OA knee compared to non-surgical management across the world, which mostly consists of studies from developed countries." This statement might be misleading to readers. It is important to clarify that this statement reflects findings from existing research rather than a conclusion drawn from your own study. Consider revising it to: "Previous literature reviews indicated that TKR surgery is often considered a cost-effective treatment method for OA knee compared to non-surgical management, primarily based on studies conducted in developed countries worldwide."**

We have revised it to “Our literature review suggests that TKR surgery is often considered a cost-effective treatment method for OA knee compared to the non-surgical management, primarily based on studies conducted in the developed countries”

**7. Needs some adjustments and format change for improved consistency, clarity and readability
(1) Figure 1, particularly the "Reports not retrieved" section is incomplete.
(2) Several typo errors and instances of missing punctuation have been noticed, such as in Line 324. Kindly make the necessary corrections.
(3) For Figure 2, it is recommended to use the currency format ($##,###).**

Thank you for the suggestions. The changes have been incorporated accordingly.

**8. Did you consult with a librarian to develop your search terms? If so, please mention in your methods.**

We did not consult a librarian during the development of search terms or at any point of the review.
